# Canine Hereditary Ataxia in Old English Sheepdogs and Gordon Setters Is Associated with a Defect in the Autophagy Gene Encoding *RAB24*

**DOI:** 10.1371/journal.pgen.1003991

**Published:** 2014-02-06

**Authors:** Caryline Agler, Dahlia M. Nielsen, Ganokon Urkasemsin, Andrew Singleton, Noriko Tonomura, Snaevar Sigurdsson, Ruqi Tang, Keith Linder, Sampath Arepalli, Dena Hernandez, Kerstin Lindblad-Toh, Joyce van de Leemput, Alison Motsinger-Reif, Dennis P. O'Brien, Jerold Bell, Tonya Harris, Steven Steinberg, Natasha J. Olby

**Affiliations:** 1 Department of Clinical Sciences, College of Veterinary Medicine, North Carolina State University, Raleigh, North Carolina, United States of America; 2 Bioinformatics Research Center, North Carolina State University, Raleigh, North Carolina, United States of America; 3 Laboratory of Neurogenetics, National Institute on Aging, Bethesda, Maryland, United States of America; 4 Broad Institute of MIT and Harvard, Cambridge, Massachusetts, United States of America; 5 Department of Clinical Sciences, Tufts Cummings School of Veterinary Medicine, North Grafton, Massachusetts, United States of America; 6 Department of Population Health and Pathobiology, College of Veterinary Medicine, North Carolina State University, Raleigh, North Carolina, United States of America; 7 Science for Life Laboratory, Department of Medical Biochemistry and Microbiology, Uppsala University, Uppsala, Sweden; 8 Center for Comparative Medicine and Translational Research, North Carolina State University, Raleigh, North Carolina, United States of America; 9 Department of Veterinary Medicine & Surgery, College of Veterinary Medicine, University of Missouri, Columbia, Missouri, United States of America; 10 VCA Veterinary Referral Associates, Gaithersbrug, Maryland, United States of America; University of Bern, Switzerland

## Abstract

Old English Sheepdogs and Gordon Setters suffer from a juvenile onset, autosomal recessive form of canine hereditary ataxia primarily affecting the Purkinje neuron of the cerebellar cortex. The clinical and histological characteristics are analogous to hereditary ataxias in humans. Linkage and genome-wide association studies on a cohort of related Old English Sheepdogs identified a region on CFA4 strongly associated with the disease phenotype. Targeted sequence capture and next generation sequencing of the region identified an A to C single nucleotide polymorphism (SNP) located at position 113 in exon 1 of an autophagy gene, *RAB24*, that segregated with the phenotype. Genotyping of six additional breeds of dogs affected with hereditary ataxia identified the same polymorphism in affected Gordon Setters that segregated perfectly with phenotype. The other breeds tested did not have the polymorphism. Genome-wide SNP genotyping of Gordon Setters identified a 1.9 MB region with an identical haplotype to affected Old English Sheepdogs. Histopathology, immunohistochemistry and ultrastructural evaluation of the brains of affected dogs from both breeds identified dramatic Purkinje neuron loss with axonal spheroids, accumulation of autophagosomes, ubiquitin positive inclusions and a diffuse increase in cytoplasmic neuronal ubiquitin staining. These findings recapitulate the changes reported in mice with induced neuron-specific autophagy defects. Taken together, our results suggest that a defect in *RAB24*, a gene associated with autophagy, is highly associated with and may contribute to canine hereditary ataxia in Old English Sheepdogs and Gordon Setters. This finding suggests that detailed investigation of autophagy pathways should be undertaken in human hereditary ataxia.

## Author Summary

Neurodegenerative diseases are one of the most important causes of decline in an aging population. An important subset of these diseases are known as the hereditary ataxias, familial neurodegenerative diseases that affect the cerebellum causing progressive gait disturbance in both humans and dogs. We identified a mutation in *RAB24*, a gene associated with autophagy, in Old English Sheepdogs and Gordon Setters with hereditary ataxia. Autophagy is a process by which cell proteins and organelles are removed and recycled and its critical role in maintenance of the continued health of cells is becoming clear. We evaluated the brains of affected dogs and identified accumulations of autophagosomes within the cerebellum, suggesting a defect in the autophagy pathway. Our results suggest that a defect in the autophagy pathway results in neuronal death in a naturally occurring disease in dogs. The autophagy pathway should be investigated in human hereditary ataxia and may represent a therapeutic target in neurodegenerative diseases.

## Introduction

The hereditary ataxias are an important, heterogeneous group of movement disorders unified by the presence of degeneration of the cerebellar cortex, and in particular of the Purkinje neurons [1]–[5]. They may be inherited as autosomal dominant (also known as the spinocerebellar ataxias or SCAs), recessive, X linked and mitochondrial traits with the autosomal dominant SCAs representing the most common group of ataxias in humans [3], [4]. Associated mutations range from possible toxic gain of function mechanisms in polyglutamine diseases [6], to ion chanelopathies such as the proposed calcium channel dysfunction in SCA6 [7], to abnormalities in growth factors such as FGF14 in SCA27 [8], and to structural proteins such as β-III spectrin in SCA5 [9]. While over 50 different genetic loci have been shown to be associated in humans, in 20–40% of patients, the genetic cause remains elusive [6]. Purebred dogs suffer from comparable neurodegenerative diseases affecting the cerebellar cortex referred to as cerebellar cortical degeneration, cerebellar abiotrophy or canine cerebellar or hereditary ataxia. Currently, hereditary cerebellar degenerative disorders have been described in over 20 breeds of dog [10], [11] with many more sporadic cases reported. In most dog breeds, the disorder causes slowly progressive degeneration of the cerebellar cortex, with dramatic Purkinje neuron loss resulting in a progressive gait dysfunction [10], [11]. Forms that primarily target the granular layer or produce ataxia without cell loss are also reported [12]–[18]. The canine form of the disease targets the cerebellum primarily and is most commonly inherited as a fully penetrant autosomal recessive trait [11], [13], [19], [20]. Onset may be neonatal [13], juvenile [21] or even older [22]. The genetic cause of three of these canine disorders has been described and include a mutation in *SEL1L*, a protein important in endoplasmic reticulum associated protein degradation in the Finnish hound [23], *SPTBN2* gene encoding β-III spectrin in the beagle [24], and *GRM1*, the metabotropic glutamate 1 receptor in the Coton de Tulear [25]. Autosomal recessive cerebellar degenerative disorders have been described in the Old English Sheepdog [20] and the Gordon Setter [26], [27]. The clinical phenotype is identical in both breeds with an onset of cerebellar ataxia first noted in juvenile to young adult dogs aged from six months to four years. Dogs develop pronounced hypermetria, a truncal sway and intention tremor, and signs progress to cause severe gait disturbances. Cerebellar atrophy can be identified by magnetic resonance imaging (MRI) [28] and histopathological findings include loss of Purkinje cell, granule cell and molecular layer neurons causing atrophy of the cerebellar cortex. In more detailed work on Gordon Setters, profound changes in cerebellar neurotransmitter levels and synapses have been described [29], along with the development of Purkinje neuron axonal spheroids [30].

The aim of this project was to investigate the genetic cause of hereditary ataxia in Old English Sheepdogs. To that effect, we genotyped an extended family of Old English Sheepdogs that suffer from canine hereditary ataxia, and identified a chromosomal locus associated with the trait using linkage and genome-wide association analyses. Sequence capture of the associated region was performed to facilitate fine mapping and this identified a mutation that segregated with the condition. Dogs from other breeds affected with cerebellar degeneration were also screened for the mutation and the identical mutation was found in Gordon Setters with canine hereditary ataxia.

## Results

### Linkage analysis in Old English Sheepdogs links disease trait to CFA4

#### Microsatellite genotyping of Old English Sheepdogs

**Figure 1:**
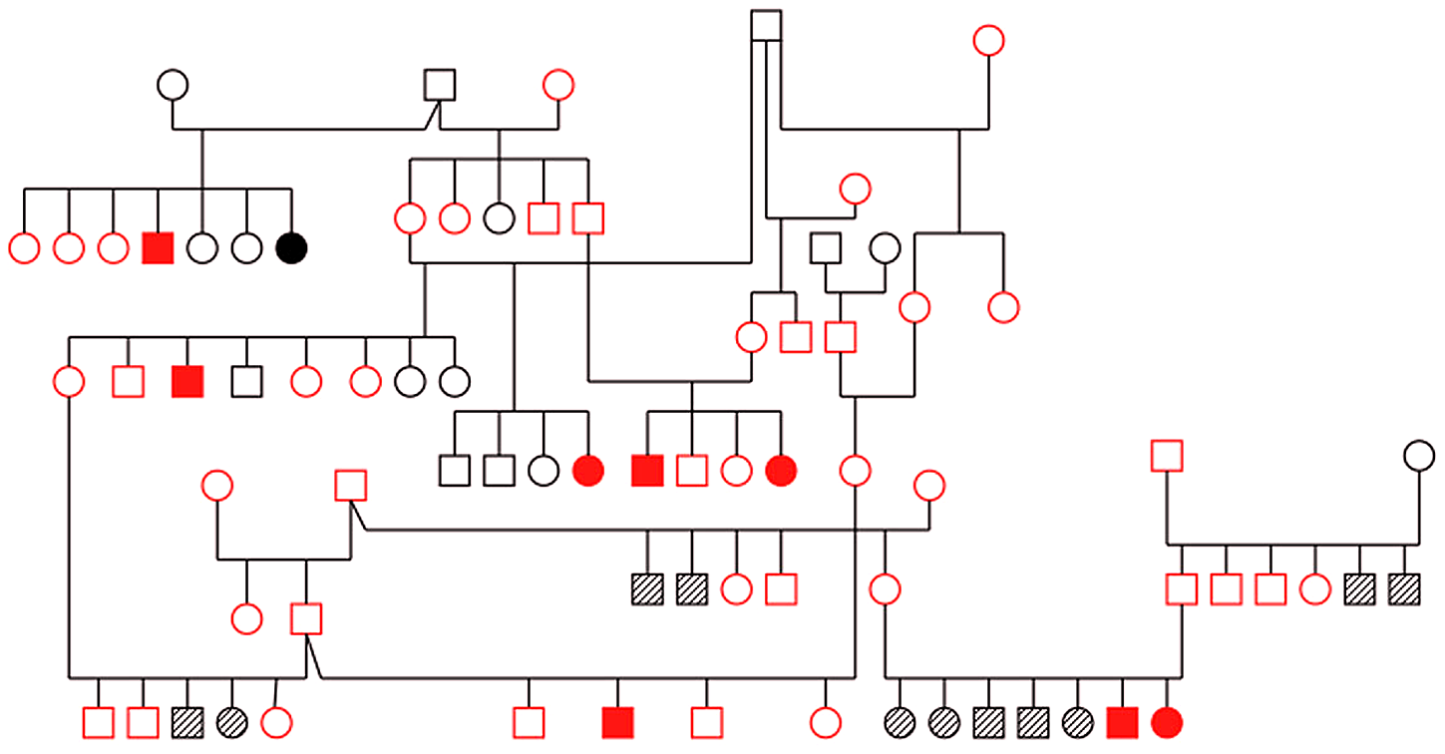
Pedigree of family of Old English Sheepdogs genotyped with microsatellite markers for linkage analysis. Red: genotyped. Solid symbol: cases, open symbol: controls, hashed symbol: unknown.

Blood samples were obtained from families of affected Old English Sheepdogs by solicitation through the Old English Sheepdog Club of America. Samples from 343 dogs including 15 affected dogs were collected. All affected dogs exhibited slowly progressive cerebellar ataxia (Video S1) and the phenotype was confirmed with histopathology in seven cases. Forty-eight related Old English Sheepdogs, including eight affected dogs (Figure 1) were selected for genome-wide genotyping [31]. Once uninformative markers were excluded, 264 markers were available for linkage analysis.

#### Linkage analysis

**Figure 2:**
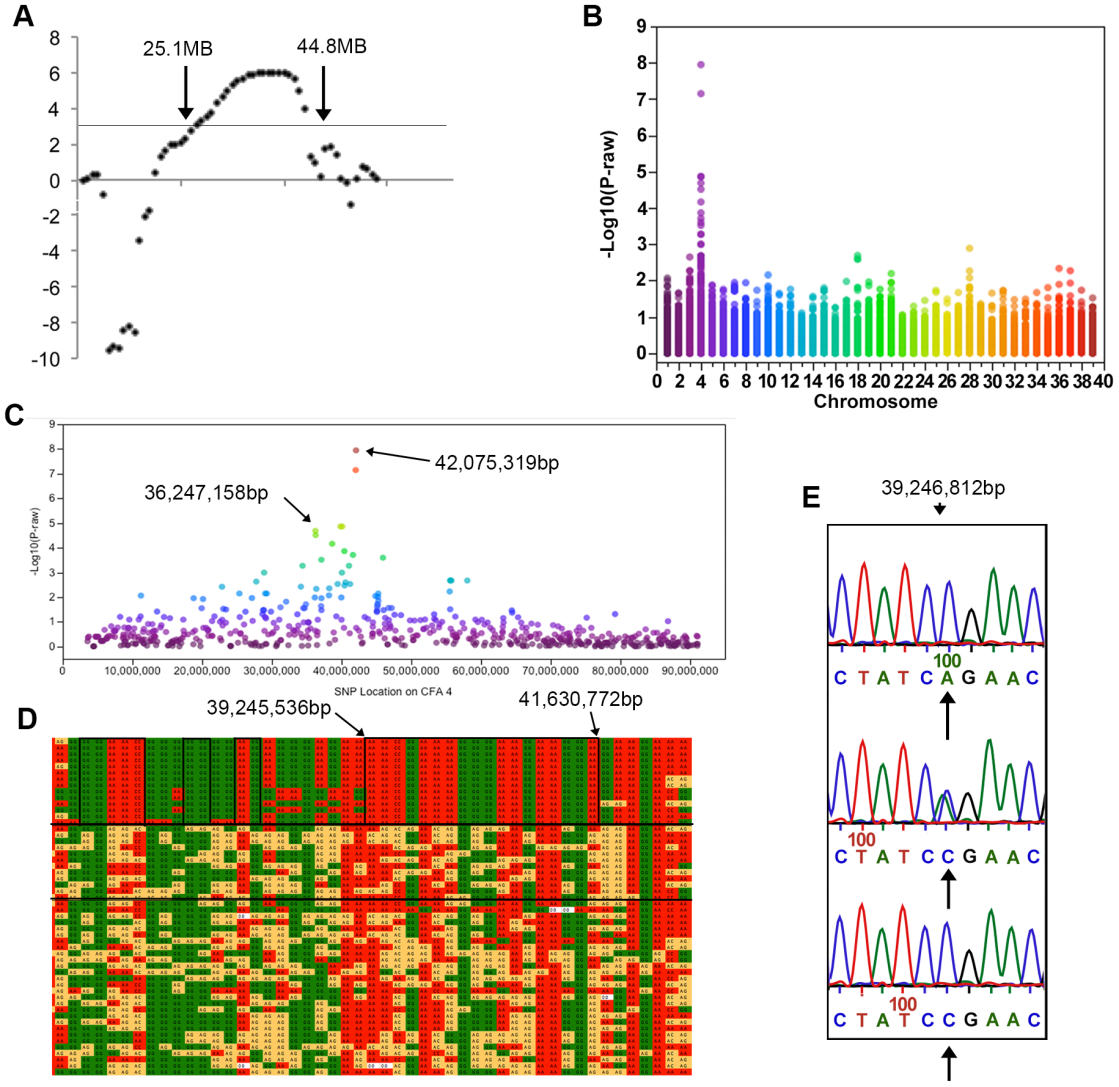
Results of genotyping, linkage and genome-wide association analyses. All coordinates refer to CanFam 2. a) LOD scores on CFA 4. LOD scores >3 were found between markers 408 and REN74B1 located at 25.1 and 44.8 MB respectively. b) A Manhattan plot of −log_10_ p-value against chromosome. This genome-wide association study using 12,986 SNPs showed significant association on CFA4. c) On CFA4 SNPs with significant association after Bonferroni correction were located between 36 and 42 MB. d) The SNP genotypes are shown in a chart. Each row is a different individual and each column is a different SNP. Solid green and red boxes indicate homozygosity while yellow boxes indicate heterozygosity. The genotype of cases is displayed in the top segment of the chart, their parents are shown in the middle section between the two solid, horizontal black lines, and the remaining phenotypically normal dogs in the bottom segment of the chart. A large region of homozygosity extends from 35,402,483 bp to 41,772,116 bp in all affected dogs and smaller regions in which the affected dogs are identical and different from the normal dogs are indicated by black boxes. * lies over the two most significant SNPs from the GWAS. e) Sequence of a portion of *RAB24* showing the A>C mutation. Arrows under the traces indicate the target nucleotide. Normal wild type dogs are shown in the top electropherogram, heterozygotes in the middle electropherogram and homozygotes for the mutation in the bottom electropherogram.

Multipoint linkage analysis was performed using the lm_Bayes program of Morgan (v2.8.1), a software package specifically designed to manage inbred populations [32]. Based on published pedigree analysis, a highly penetrant autosomal recessive mode of inheritance was assumed [20]. Preliminary linkage analysis revealed a region on CFA4 that had LOD scores of greater than 3 between markers FH3310 and FH2776. As these markers were over 17 MB apart, eight additional microsatellites within this region were identified from the UC Davis Canine Genetic Linkage map and genotyped in the same individuals. This resulted in a maximum LOD score of 6 on CFA4 and LOD scores greater than 3 extending from marker 408 located at 25,086,893 bp to REN74B13 located at 44,763,308 bp (Figure 2a). Evaluation of the haplotypes in the region failed to identify clear segregation with disease. This region was gene dense but did not contain genes already known to be associated with hereditary ataxia.

### A Genome-wide association study confirms the linkage analysis results and identifies a region of homozygosity

#### SNP genotyping of Old English Sheepdogs

In order to provide greater resolution of the trait-linked locus, SNP genotyping was performed in 54 Old English Sheepdogs, the original 48 used in the linkage analysis and an additional six affected dogs for a total of 14 affected dogs. Dogs were genotyped using the Illumina Infinium CanineSNP20 Beadchip (22 dogs) and CanineHDBeadchip (32 dogs) (Illumina; San Diego, CA). All dogs had a greater than 96% call rate. For the purpose of the GWAS, only SNPs common to both the SNP20 and the HD Beadchips were used. SNPs with a minor allele frequency of less than 1% were pruned, leaving a final data set consisting of 12,986 SNPs common to both SNP chips. A case-control association study of 14 (10 male, 4 female) cases and 40 controls (17 male, 23 female) using PLINK [33] detected a region of significant association on CFA4 with two SNPs passing Bonferroni correction at positions 42,075,319 bp (p_raw_ = 1.8×10^−8^, p_bonferroni_ = 1.32×10^−4^) and 42,030,138 bp (p_raw_ = 6.68×10^−8^, p_bonferroni_ = 8.67×10^−4^) (Figures 2b and c). Four additional SNPs were detected in the region on CFA4 spanning 36,247,158 bp to 40,088,807 bp based on CanFam2 (Figure 2c). In spite of the dogs coming from the same pedigree, the genomic inflation factor λ was 1, suggesting that family structure did not cause an inflation in the association test results. However, in order to control for false positive associations resulting from evaluation of related dogs, a family-based association test was also performed. A significant association using FBAT [34] was again detected on CFA4 with SNPs spanning 32,042,029 bp to 42,030,138 bp. SNPs at 42,075,319 bp and 42,030,138 bp again had the highest significance at (p_raw_ = 1.1×10^−5^, p_bonferroni_ = 1.1×10^−3^).

#### Haplotype analysis

Genotype data was visually inspected for regions of homozygosity shared only in the affected dogs. There was a region of homozygosity extending from 35,402,483 bp to 42,752,780 bp in all affected dogs (Figure 2d). Within that region there was one 3 MB block extending from 39,245,536 bp to 41,630,722 bp, and several smaller blocks that were identical in affected dogs (Figure 2d).

### Targeted sequence capture identifies six non-synonymous exonic SNPs that segregate with phenotype

Targeted sequence capture was performed to sequence the entire genomic region from 34 MB to 46 MB on CFA4, thereby covering the whole homozygous region. Six dogs were sequenced, three cases, and three controls, including two obligate heterozygotes and one unrelated, phenotypically normal dog. A Roche NimbleGen array (Madison, WI) was designed to provide coverage of 95.7% of the region using unique probes. The design was reviewed to ensure that all predicted and known gene exons had adequate coverage. Following next-generation sequencing on Illumina's Hi-Seq 2000 machine (Illumina; San Diego, CA), approximately 96% of the 12,000,000 targeted bases had at least 2× coverage with approximately 72% having at least 30× coverage. Quantitative PCR confirmed that the region of interest was enriched appropriately.

Data processing using the Genome Analysis Toolkit (GATK) [35] revealed 40,711 total variants (32,475 SNPs and 8,236 indels). After filtering the total variants for known SNPs and those present in all six sequenced dogs, 28,061 variants remained of which only 288 were present in coding regions. Screening of these 288 variants revealed nine SNPs that segregated in an autosomal recessive inheritance pattern, six of which resulted in an amino acid change and were considered potential causative mutations for the disease trait. The six SNPs were located in the genes *RGR*, *RAB24*, *NSD1*, *GPRIN1*, and *CDHR2* (Table S1).

### Re-sequencing and genotyping of a larger population of dogs identifies one SNP in *RAB24* that segregates with phenotype

The six SNPs identified as mutations of interest were verified by Sanger sequencing in the six dogs initially sequenced. Additional cases and controls were genotyped on the six SNPs (Table S2). SNPs in *RGR*, *GPRIN1*, and *CDHR2* did not segregate with phenotype in this larger population and were eliminated from further analysis. The two SNPs present in *RAB24* and *NSD1* showed segregation in the cases and controls consistent with an autosomal recessive mode of inheritance (Table S2). When these two SNPs were tested for association with the trait in the same 53 dogs as the GWAS, the p-values were both highly significant at p_bonferroni_ = 5.7×10^−9^. When additional dog breeds were tested (Table S3), the *NSD1* SNP was present in the heterozygous state in three neurologically normal Labrador Retrievers (from a group of 7 dogs) and two neurologically normal Standard Poodles (from a group of 7 dogs). Cerebellar degeneration has not been reported in Standard Poodles but has been reported in Labrador Retrievers [36],[37]. However, an affected Labrador retriever was genotyped for this SNP and it was not present in the affected dog (see results below). The presence of this SNP in five of 14 dogs from two breeds implies a relatively high prevalence of this polymorphism within these breeds. The lack of reports of hereditary ataxia in one of these breeds and the absence of this SNP in an affected dog from the other breed make it unlikely that it is a pathogenic mutation and focused our attention on *RAB24*.

The *RAB24* SNP polymorphism was an A to C transversion located at position 113 in the first of its eight exons (Figure 2e) and it produced an amino acid change from glutamine (Q) to proline (P) at position 38. In order to investigate the hypothesis that the Rab24 p.Q38P change was the causative mutation for hereditary ataxia in Old English Sheepdogs, a total of 376 Old English Sheepdogs were genotyped, all of which came from case blood lines, including the 14 cases used in the GWAS and an additional six cases. Of these dogs, all 20 confirmed cases were homozygous for the alternate allele. 109 controls were heterozygotes, four controls were homozygous for the alternate allele while the remainder were homozygous for the wild type allele. This cohort of dogs had been sampled approximately 20 years previously and at the time, their owners provided the dogs' phenotype. All dogs were old enough to be expected to exhibit neurological signs if affected. Of the four dogs that were reported as normal but exhibited the case genotype, two were littermates of affected dogs, with one confirmed affected parent and one parent confirmed as a carrier. The remaining two dogs were descended from parents who were carriers. Physical examinations were not performed on these dogs by a veterinarian and so their phenotype could not be confirmed.

**Figure 3:**
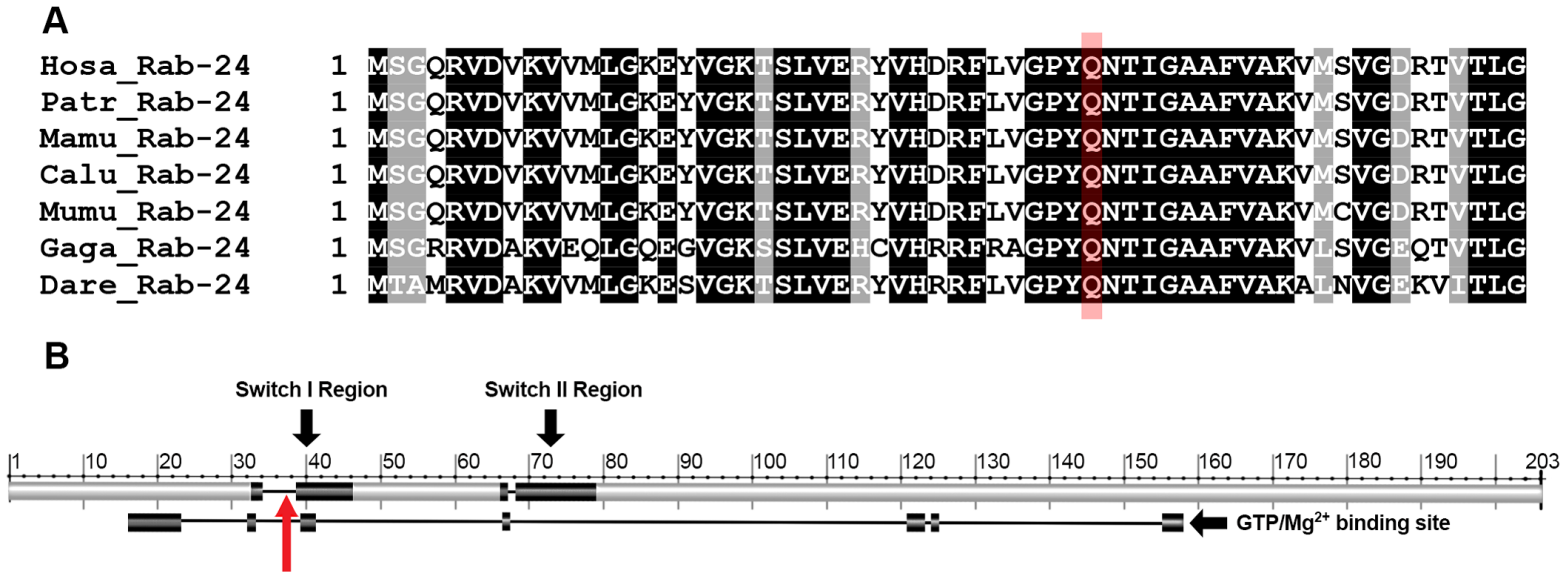
The Rab24 protein and mutation location. a) ClustalW multiple protein alignment of the region of Rab24 containing the mutation. The target glutamine is highlighted in red. Sequences for humans (Hosa), chimpanzees (Patr), rhesus macaque (Mamu), wolf (Calu), mouse (Mumu), chicken (Gaga) and zebrafish (Dare) are shown. A black background indicates 100% homology in these species; the target glutamine lies within a region of 14 highly conserved amino acids. b) Proposed switch I and II regions are shown superimposed on the Rab24 protein and the GTPase/Mg^2+^ binding site is shown below the protein. The mutation at the 38^th^ amino acid lies within switch I region and the GTPase/Mg^2+^ binding site (red arrow).

The effect of the Rab24 p.Q38P change on protein function was investigated using two different homology-based on-line tools, Polyphen-2 [38] and SIFT [39]. Both predicted that the change would probably damage protein function, with a score of 0.989 for Polyphen-2 (sensitivity: 0.72 and specificity: 0.97) and 0.01 for SIFT. The Rab24 protein is a GTPase from the large Rab protein family important in vesicle trafficking, endocytosis and exocytosis [40]. Several Rab proteins, including Rab24, have now been shown to play a vital role in autophagy [41], the process by which proteins and organelles are moved to lysosomes for degradation. Domains known to be important to Rab protein function include the nucleotide binding and Mg^2+^sites necessary for GTPase activity; two switch regions play a vital role in facilitating GTPase activity. The p.Q38P change lies in a highly conserved amino acid (Figure 3a) located within the putative switch I region, suggestive of an effect on GTP binding (Figure 3b).

An additional 254 DNA samples from Old English Sheepdogs were obtained from the Orthopedic Foundation for Animals Inc. (OFA) CHIC DNA repository. Detailed phenotypic information was not available on these dogs although none were reported to have canine hereditary ataxia on health questionnaires completed by owners at the time of DNA submission. This cohort of dogs came from approximately 70 different breeding kennels. These samples were genotyped in order to determine the prevalence of the mutation in a wider population of dogs. Twenty-eight of these dogs were heterozygous while the remainder was homozygous for the reference allele, giving an overall alternate allele prevalence in the original pedigrees studied and the random selection of dogs (a total of 630 dogs) of 14.3%.

To test for the potential of the mutation to discriminate cases and controls, receiver operating characteristic (ROC) curve analysis was performed in the total population of Old English Sheepdogs genotyped. The area under the curve was 99.5% (S.E. = 0.0023, 95% confidence interval = 0.9909–0.9999), which was highly statistically significant (p<0.001).

### Genotyping of additional affected dogs of different breeds reveals the same *RAB24* mutation in Gordon Setters with hereditary ataxia

Hereditary ataxia is not unusual in dogs and has been reported in numerous breeds. In order to determine whether the p.Q38P mutation was found in affected dogs from other breeds, DNA samples from a Dalmatian, a beagle (both confirmed by neurological evaluation and the presence of cerebellar atrophy on MRI of the brain), 2 Rhodesian Ridgebacks (necropsy confirmed), 2 Gordon Setters (one of which was necropsy confirmed and the other confirmed by neurological evaluation and the presence of cerebellar atrophy on MRI of the brain), 2 Scottish Terriers (one MRI and one necropsy confirmed) and a Labrador retriever (diagnosed by neurological evaluation and clinical history only) were genotyped for the *RAB24* and the *NSD1* SNPs. In addition, all exons of the *RAB24* gene were sequenced in these affected dogs. The alternate allele for *RAB24* or *NSD1* was not present in any of the breeds except the affected Gordon Setters. Two heterozygous synonymous SNPs were identified in exon 3 of *RAB24* in the beagle which were also present in the Old English Sheepdog sequencing data. Heterozygous non-exonic SNPs were also identified in the Dalmatian. None of these were consistent with the mode of inheritance or pathologic in nature.

A total of 18 affected Gordon Setters were then genotyped, all of which were homozygous for the *RAB24* mutation. DNA from ten of these cases was obtained from archived material from a research dog colony [19]. An additional 82 normal Gordon Setters were genotyped from Scandinavia and the US and 24 dogs were heterozygotes and 58 were homozygous for the wild type allele. None were homozygous for the alternate allele. Excluding the 10 cases that were archival material and therefore not part of the general breeding population, there was an alternate allele frequency of 22.2%. The phenotype of cerebellar degeneration in Gordon Setters has been described in detail [19], [26], [27] and is identical to the description of Old English Sheepdogs in terms of age of onset and progression of signs. To compare the genetic background of Gordon Setters and Old English Sheepdogs further, a cohort of seven affected and 26 control Gordon Setters were genotyped on the Illumina Infinium Canine HDBeadchip and the haplotypes on CFA4 were compared to those of the Old English Sheepdogs. This revealed an identical region of homozygosity extending from 39,245,536 bp to 41,172,873 bp in affected dogs from both breeds, including the *NSD1* mutation also identified in the region (Figure S1), suggesting the mutation dates back to a common European ancestral dog population, from which these two separate breeds were founded. This region contains 29 genes inferred from human sequence data (Table S4). When targeted sequencing was performed in the Old English Sheepdogs, mean sequencing coverage of this region of shared homozygosity was 47×, making it unlikely that additional variants were missed. These findings are supportive of the hypothesis that the *RAB24* mutation is the causative mutation of hereditary ataxia in these two breeds of dog.

In order to determine whether this mutation was present in other breeds of dog representing diverse ancestral lineage, DNA samples were collected from at least eight individuals from breeds in each of 10 breed clusters reported as related ancestrally [42], [43]. A total of 194 individuals from 43 different breeds were genotyped (Table S3). All additional breeds were homozygous for the wild type allele on the *RAB24* SNP.

### RNA sequencing and qRT-PCR do not show a change in level of expression of *RAB24*

Expression levels of *RAB24* in the cerebellum were compared between case (n = 4) and control (n = 2) dogs by qRT-PCR. There was no significant difference in level of *RAB24* expression between cases and controls (p-value = 0.71). The A>C change in the *RAB24* transcript was present in all affected dogs and absent in the control dogs. The predicted exon/intron boundaries were also confirmed.

### Histopathology, immunohistochemistry and ultrastructural evaluation reveal Purkinje neuron loss, with associated axonal spheroids containing autophagosomes and staining positive for ubiquitin

Samples of brain from eight affected Old English Sheepdogs ranging in age from 2.5 to 13 years, and one affected 2.5-year-old Gordon Setter, and from 10 neurologically normal, age matched dogs were fixed in 10% neutral buffered formalin and embedded in paraffin for histological evaluation. Six of the eight Old English Sheepdogs were examined at the University of Pennsylvania in 1999 and the paraffin embedded blocks were retrieved from the archives. In the remaining two Old English Sheepdogs, the brains were harvested by a local veterinarian following euthanasia, placed in formalin and shipped to NCSU for paraffin embedding. The Gordon Setter was euthanized at NCSU and the brain was removed immediately and placed in formalin within 30 minutes of euthanasia. Sections were stained with hematoxylin and eosin, Periodic Acid Schiff (PAS, to evaluate glycogen storage products), Bielschowsky silver stain (to evaluate axonal processes), and luxol fast blue (to evaluate myelination). Immunohistochemical staining was performed for GFAP, ubiquitin, and Rab24. Samples of cerebellum from the affected Gordon Setter were fixed in McDowell's and Trump's 4F:1G fixative and processed for electron microscopy.

**Figure 4:**
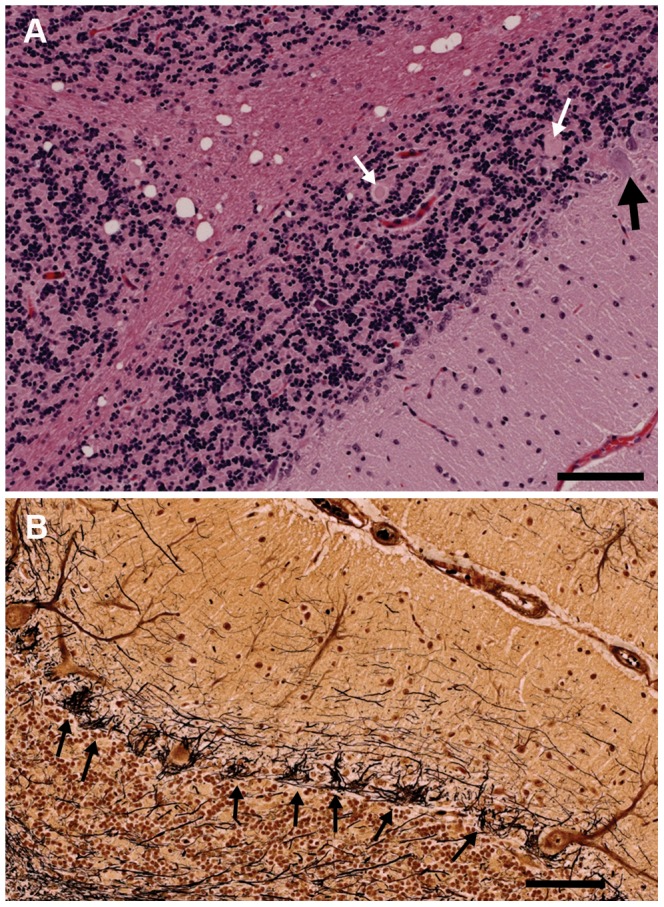
Sections of the cerebellum from an affected 2.5-year-old Gordon Setter stained with hematoxylin and eosin (a) and Bielschowsky (b). a) The three cortical layers and cerebellar cortical white matter are shown. There is dramatic loss of Purkinje neurons with only one Purkinje neuron (black arrow) visible at the junction of the granular and molecular layers. Axonal spheroids can be seen within the granular layer (white arrows) and there is marked vacuolation of the white matter. Bar = 100 µm. b) The Bielschowsky stain highlights the processes of basket cells around Purkinje neurons, thus emphasizing the loss of Purkinje neurons (black arrows).

Pathological changes were largely restricted to the cerebellum, with the majority of changes affecting the cerebellar cortex. There was dramatic loss of Purkinje neurons, with atrophy of the molecular and granular layers (Figure 4a). Vacuoles were visible in the white matter throughout the cerebellum and axonal spheroids were identified in the granular layer (Figure 4a). In addition, there were vacuoles in the cerebellar peduncles, the vestibular and cochlear nuclei and the nucleus of the dorsal trapezoid body. There were minimal changes in the cerebellar nuclei. The Bielschowsky stain highlighted the processes of basket cells and the lack of Purkinje neurons (Figure 4b). There was no evidence of glycogen accumulation on PAS stained sections and the luxol fast blue staining confirmed the presence of myelin around the axonal spheroids identified in the granular layer. Immunostaining for GFAP showed increased astrocytic expression and highlighted mild to moderate astrocytosis.

**Figure 5:**
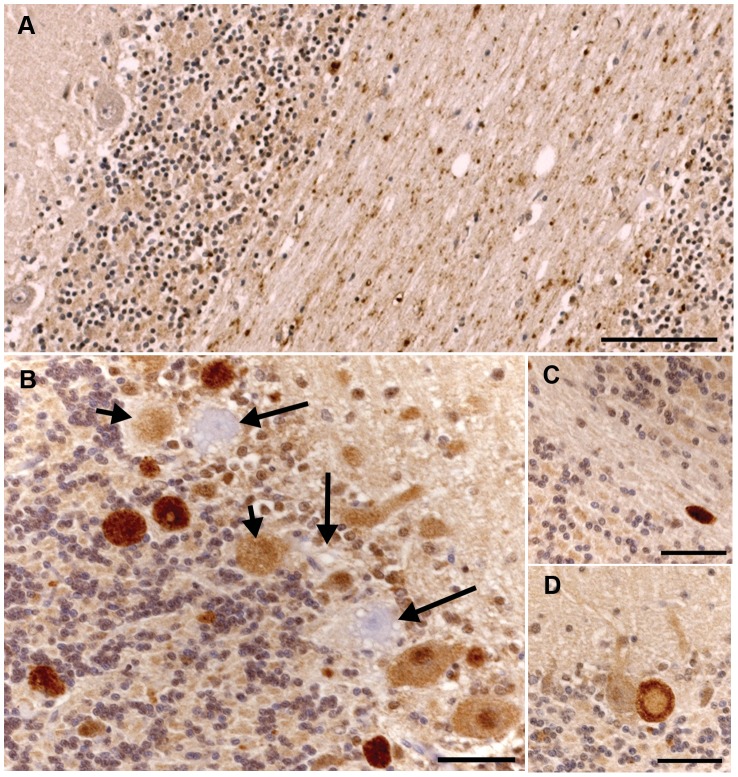
Ubiquitin immunohistochemical staining of the cerebellum of an unaffected 11-year-old Dachshund and an affected 2.5-year-old Gordon Setter. a) There is granular ubiquitin positive material in the white matter tracts of the cerebellum in this unaffected dog. The Purkinje neurons are negative. Bar = 100 µm. b) There are multiple large, intensely ubiquitin positive circular bodies in the granular layer and at the junction of the molecular and granular layers in this affected Gordon Setter. The ubiquitin staining has a punctate character within these bodies. In addition, there are Purkinje and molecular neurons with diffuse cytoplasmic and nuclear ubiquitin staining. Note also the three highly vacuolated Purkinje neurons that are negative for ubiquitin (long arrows). Two of these degenerating Purkinje neurons have ubiquitin positive circular bodies immediately adjacent to them (short arrows). These circular bodies correlated to axonal spheroids on the hematoxylin and eosin stains and, as shown in figure 5d, appeared to connect directly to Purkinje neurons in some instances. Bar = 50 µm. c) An example of a large ovoid ubiquitin positive body in the cerebellar white matter of the 2.5-year-old affected dog. Bar = 50 µm. d) A ubiquitin positive body lies immediately adjacent to (and apparently connecting with) a Purkinje neuron and has a less intensely staining center. Bar = 50 µm.

**Figure 6:**
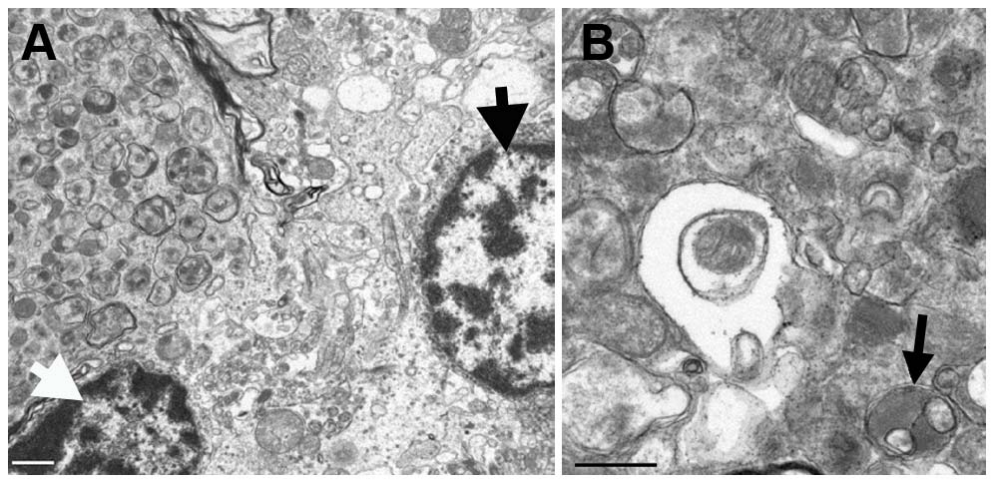
Transmission electron micrographs of the cerebellum of an affected Gordon Setter. a) A partially-captured axonal spheroid, located at the junction of the Purkinje and granular layers of the cerebellum, packed with late autophagosomes, is visible in the upper left hand side of the image and it is closely associated with a Purkinje neuron (thick black arrow) and a granular neuron (thick white arrow). Bar = 1 µm. b) At higher magnification, the wide array of morphologies of autophagosomes can be seen along with some more normal appearing mitochondria. Autophagosomes typically have a double membrane and an example of a degradative autophagosome is illustrated here (black arrow). Bar = 0.5 µm.

Ubiquitin immunostaining of controls revealed granular accumulations of ubiquitin positive material in the white matter, the density of which increased with age (Figure 5a). In older control dogs, some positive staining was also seen in the granular layer and very fine positive granules were found at the junction of the molecular and granular layers around Purkinje neurons. In cases, the ubiquitin positive staining within the white matter was comparable to the age matched controls. However, there were also multiple, large ovoid bodies containing ubiquitin positive material staining in a punctate pattern, lying within the granular layer, at the junction of the granular and molecular layers, and in the cerebellar white matter (Figure 5b and c). In some instances, these ubiquitin positive bodies appeared to be emanating from a Purkinje neuron and to co-localize with axonal spheroids seen on the hematoxylin and eosin stained sections (Figure 5b and d). Ubiquitin positive bodies were limited to the cerebellum of cases, with no inclusions seen elsewhere in the brain. In addition, some Purkinje neurons, molecular layer neurons and Golgi neurons within the granular layer had strong diffuse cytoplasmic ubiquitin staining (Figure 5b). The axonal spheroids were examined on electron microscopy and were packed with organelles such as mitochondria and the Golgi apparatus, and vesicular structures, many of which had the classic double membrane of autophagosomes (Figure 6).

**Figure 7:**
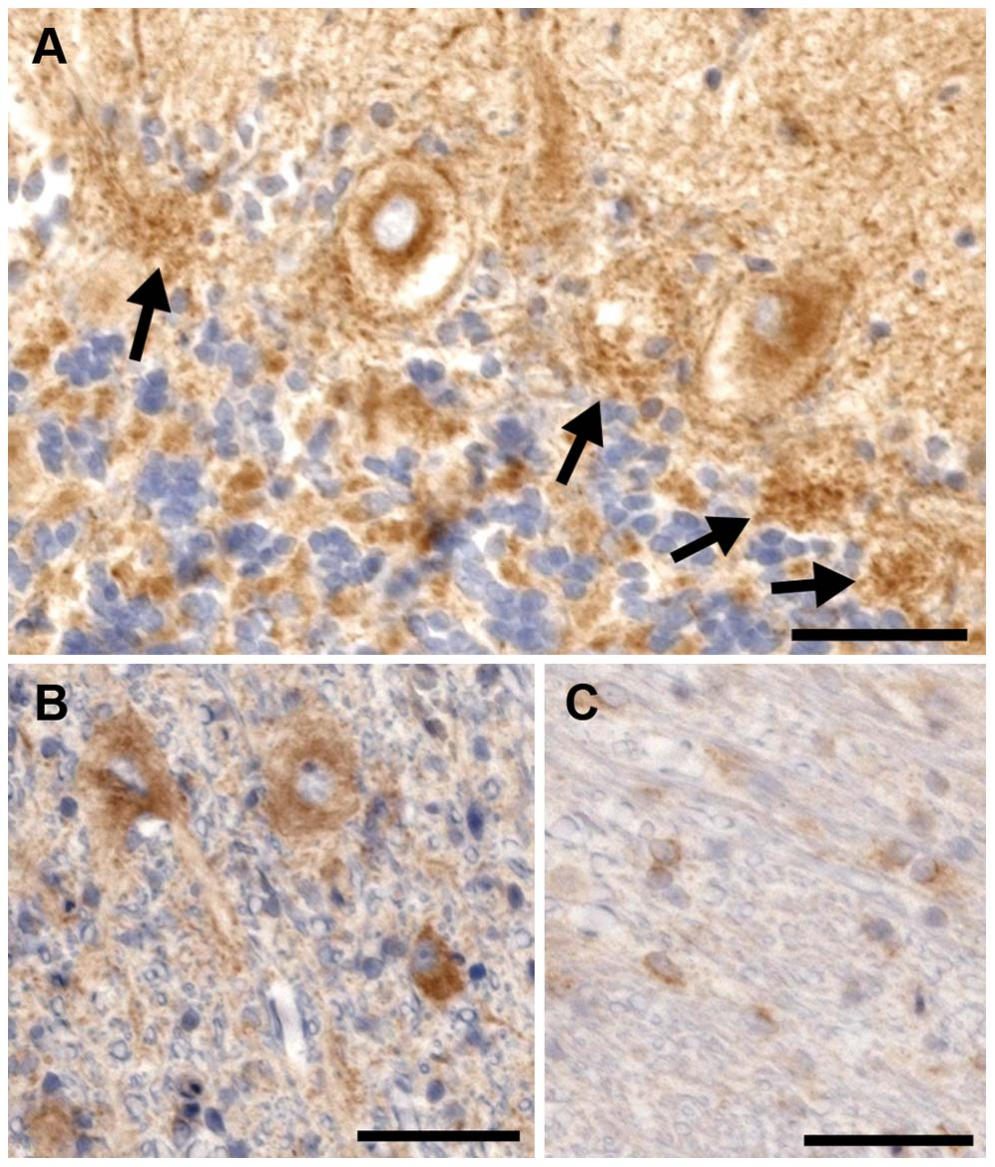
Rab24 immunohistochemical staining of the cerebellum of an affected dog. a) There is intense eccentric perinuclear staining of the Purkinje cells and the processes of the basket cells. The basket cell staining can still be seen in places where the Purkinje neurons have died (arrows). b) The neurons in the deep cerebellar nuclei all exhibit cytoplasmic staining for Rab24. c) Cells stained positive within the white matter of the cerebellum. Due to their morphology, these cells are believed to be oligodendrocytes. Bar = 100 µm in all images.

There was no significant difference in the intensity or pattern of Rab24 immunostaining in the cerebellum of cases and controls. There was positive staining within the cytoplasm of Purkinje neurons; the staining was granular and located in an eccentric perinuclear position as described in cell culture studies [44]–[46] (Figure 7a). The terminal dendrites of the basket cells on Purkinje neurons stained intensely (Figure 7a). In addition, neurons in the molecular layer, and the granular layer stained positively, as did the neurons of the deep cerebellar nuclei (Figure 7b) and oligodendrocytes (Figure 7c) throughout the white matter of the cerebellum. Axonal spheroids were negative for Rab24.

## Discussion

### Hereditary ataxia in Old English Sheepdogs and Gordon Setters is associated with a mutation in *RAB24*

The results of our work implicate a founder mutation in the GTPase Rab24 as the cause of autosomal recessive hereditary ataxia in both the Old English Sheepdog and the Gordon Setter although dysfunction of this protein has not been established. Genome wide linkage and association studies in a cohort of related Old English Sheepdogs identified a strong association between the disease phenotype and an approximately 12 MB region of homozygosity on CFA 4 in affected dogs. High throughput sequencing identified an A>C variant that predicted a Q>P change in the Rab24 protein. Evaluation of Gordon Setters with the same clinical phenotype revealed the same mutation in affected dogs and a shared block of homozygosity extending over 1.9 MB. While LD extends over long distances within breeds, across breeds, LD rapidly drops off. This is a characteristic resulting from two different bottlenecks in the history of domesticated dogs, the first dating back to their separation from wolves over 10,000 years ago, and the second more recent bottleneck occurring as modern breeds developed from a limited number of founder dogs only a few hundred years ago [47], [48]. These breeds of dog are placed in different ancestral clusters with Old English Sheepdogs classified as herding dogs and Setters clustering with working dogs. This long shared haplotype between the two breeds is unusual, and the implications of our findings are that this mutation dates back to a time before these two breeds of dog were developed, that the two breeds have a shared ancestry, and that this shared founder mutation is the cause of hereditary ataxia in these breeds. This has been found with other mutated canine genes, for example, the mutation in prcd-PRA [49].

Rab24 is an atypical member of the large Rab family of small GTPases [45]. These enzymes play a vital role in membranous transport within the cell allowing movement of cell organelles, and endocytosis and exocytosis (reviewed in [40], [50]). They work in concert with the SNARE proteins to bridge membranes and drive fusion. The mechanism by which they achieve this has been well characterized for certain members of the family, and depends on their GTP state, and their ability to prenylate and thus cycle on and off membranes. Rab24 is unlike the other members of the family, having poor GTPase activity, and reduced prenylation and its mechanism of action remains elusive [45]. It has been shown to localize to the cis Golgi and ER and to co-localize with autophagosomal markers such as LC3, and it is proposed to play a role in the late stages of autophagy related to the fusion of the autophagosome and lysosome [46]. Proteomic evaluation of autophagy networks also show interactions between Rab24 and other vesicle trafficking autophagic proteins [51]. While expressed at low levels in many tissues, it is expressed most highly in the brain [44] and there is evidence that it is upregulated during neuronal differentiation [45] and as a response to nerve injury [52]. There has been speculation that Rab24 dysfunction might play a role in neurodegenerative disease because mutations placed in a region known as the G2 domain that reduce the affinity of Rab proteins for GTP produce nuclear inclusions that disrupt the nuclear membrane, and stain positive for ubiquitin and Hsp70, typical of protein aggregates in polyglutamine diseases [53]. However, more recently, Rab24 has been shown to play a role in cell division [54]. The mutation described here lies in the putative switch 1 region, important for GTP binding [55]. In support of this region being functionally important, in another Rab associated disease, Griscelli Syndrome, a mutation in the switch 1 region of Rab27A results in a profound phenotype due to failure of the Rab protein to interact with its target melanophilin [56]. Mutations in *RAB7* have been associated with Charcot-Marie-Tooth type 2B neuropathy, a progressive neurodegenerative condition of the peripheral nervous system. One of the mutations described lies immediately adjacent to a highly conserved GTP binding domain [57]. Rab7 is involved in transport between endosomes and lysosomes, demonstrating the importance of subcellular trafficking in neuronal health.

There are two main systems that allow for turn-over of cellular organelles and proteins, the ubiquitin proteasome system (UPS) and autophagy. The ubiquitin proteasome system (UPS) is well described and is a system by which short-lived regulatory and misfolded proteins undergo non-lysosomal degradation [58], [59]. The UPS system has been shown to be vital to normal development of the central nervous system, to synaptic plasticity and to cellular homeostasis. Reflective of these important roles, many neurodegenerative diseases have been linked to abnormalities in the UPS system [58], [59]. More recently, attention has shifted to the role that autophagy might play in neurodegenerative disease. Autophagy is the process by which more long-lived proteins and organelles are incorporated into autophagosomes for delivery to vacuoles or lysosomes for degradation. There is abundant cross communication between the UPS and autophagy systems, with specific ubiquitinated proteins being moved to autophagosomes for disposal. As a result of this convergence of disposal pathways, dysfunction of autophagy can result in ubiquitin accumulation [60], [61] and accumulation of autophagosomes and ubiquitin positive inclusions, both evident in our affected dogs, are considered to be hallmarks of neurodegenerative diseases (reviewed in [62]). However, these findings are quite non-specific, being present in most neurodegenerative diseases.

Autophagy is believed to be vital for cell survival in several different ways. Constitutive autophagy plays an important role as a basal source of energy in cells with high metabolic needs, such as the Purkinje neuron, in the global turnover of cellular organelles and in the clearance of potentially toxic protein aggregates [62]–[65]. While there are a variety of hypotheses for the role of autophagy in neurodegenerative diseases [66]–[68], the most compelling evidence that a primary defect in autophagy can induce neurodegenerative disease was generated by two studies in which neuron specific dysfunction of two proteins involved in the formation of autophagasomes, Atg5 and Atg7, was induced in mice [65], [69]. In both studies, mice developed progressive motor incoordination and balance deficits accompanied by progressive neurodegeneration. There was dramatic loss of Purkinje neurons accompanied by axonal swellings and development of ubiquitin positive inclusions and diffuse intraneuronal ubiquitin accumulation. The clinical and neurodegenerative phenotypes of these mice are similar to the dogs in our study in which we saw dramatic Purkinje neuron loss, axonal spheroids containing autophagosomes and large ubiquitin positive inclusions in addition to more diffuse intracellular ubiquitin accumulation. However, we were unable to demonstrate an alteration in the level of expression of the *RAB24* gene or Rab24 protein by qPCR or immunohistochemically. It can be argued that Purkinje neurons are a natural target for many different pathological processes due to their high metabolic activity, requiring higher levels of protein and organelle turn over. Indeed, in hereditary ataxia affecting the Finnish Hound, a defect in *SEL1L*, a gene important in the endoplasmic reticulum associated degradation (ERAD) process that targets misfolded proteins to the UPS, causes early onset rapidly progressive Purkinje neuron loss [23]. Taken together, these two naturally occurring canine models of hereditary ataxia suggest that protein and organelle turnover is of vital importance to the maintenance of neuronal, and in particular, Purkinje neuron health. Additional *in vitro* work needs to be completed to demonstrate Rab24 dysfunction as a result of this mutation.

We have described a mutation that results in an amino acid change in Rab24, a protein as yet poorly understood, but believed to play a role in the late stages of autophagy. Our findings of Purkinje neuron degeneration in this naturally occurring canine neurodegenerative condition support the evidence that Rab24 is necessary for autophagy, and our clinical and histopathological findings are strongly reminiscent of the mouse studies demonstrating that defects in autophagy produce neurodegeneration targeting the Purkinje neuron. This finding may provide a novel tool for the investigation of the mechanisms of autophagy and defects in Rab proteins should be considered when investigating neurodegenerative diseases.

## Materials and Methods

### Study population

Old English Sheepdogs with canine hereditary ataxia were identified by referral from veterinarians, breeders and owners. DNA samples and pedigrees from these U.S. and Canadian dogs and their unaffected family members were collected. Affected status was determined by compatible clinical signs of cerebellar disease including ataxia, typical age of onset, physical exam, and neurological exam. Cerebrospinal fluid analysis was undertaken in many dogs, as well as a necropsy, when possible. All protocols were performed with approval from North Carolina State University's Institutional Animal Care and Use Committee. All normal dogs reached the age of four years of age with no evidence of clinical signs of ataxia. Dogs that could not definitively be classified as “affected” or “normal” based on collected information were classified as of “undetermined” status. DNA was extracted from whole blood using QIAamp DNA Blood Midi Kit (Qiagen; Valencia, CA), frozen tissues; DNeasy Blood and Tissue Kit (Qiagen; Valencia, CA), buccal swabs; QIAamp DNA Mini Kit (Qiagen; Valencia, CA), and from saliva using Oragene Animal kit (DNA Genotek; Kanata, Ontario). DNA concentrations were measured using a ND-1000 UV-Vis NanoDrop spectrophotometer (Thermo Scientific, Wilmington, DE).

### Linkage analysis

Related dogs were genotyped using a genome-wide panel of 311 canine microsatellite markers (representing an average of 9 MB resolution), organized into multiplex PCR groups [31]. Four fluorescent labels (FAM, VIC, NED and PET) were incorporated into the PCR primers to allow multiplexing. PCR fragments were analyzed on an ABI-3700 automated Genetic Analyzer (Applied Biosystems), and genotypes were assigned using GeneMapper v3.7 software (Applied Biosystems). Homozygous (uninformative) markers were excluded from further analysis.

#### Linkage analysis

Multipoint linkage analysis was performed using the lm_Bayes program of Morgan (v2.8.1) [32]. Dogs were assigned affected, control or unknown phenotypes, and the trait frequency was set to default at 61% for the common allele and 39% for the risk allele. Based on the pedigree analysis performed previously [20], penetrance was set at 98%. These values were varied once the linked region on CFA4 was identified to ensure that the results were robust. The location of microsatellites was determined from the online canine genetic linkage map at the University of California, Davis Veterinary Genomics Laboratory (http://www.vgl.ucdavis.edu/dogmap/). Allele calls not consistent with Mendelian inheritance were re-evaluated, and individuals with multiple Mendelian errors were removed from analysis. Results were evaluated for LOD scores greater than 3.

### GWAS

Genotyping of SNPs was performed using Illumina's Canine SNP20 (22,362 SNPs) and CanineHD (170,362 SNPs) genotyping beadchips (Illumina; San Diego, CA). Nine cases and 13 control dogs were genotyped on the SNP20 chip and five cases and 28 controls on the CanineHD chip. One control dog was genotyped on both chips, enabling the comparison of genotype calls between them. The assays were performed at the National Institutes of Health's Laboratory of Neurogenetics (Bethesda, MD) according to the manufacturer's instructions. The amplified DNA products were imaged using a BeadArray Reader (Illumina; San Diego, CA) and analyzed using Illumina's Bead Studio and Genome Studio software (Illumina; San Diego, CA).

Data was pruned such that individuals with less than a 95% call rate and those having a significant number of Mendelian errors were removed from further analysis. SNPs having a minor allele frequency of less than 1%, missing genotype calls greater than 10% and showing inconsistent calls between the two chips were also removed from further analysis. The pruned dataset was then used to perform a case-control and family-based association analysis. The PLINK toolset v1.07 (http://pngu.mgh.harvard.edu/~purcell/plink/) was used to perform data pruning and the case-control allelic and genotypic association tests using a Bonferroni correction for multiple comparisons [33]. As most of the Old English Sheepdogs genotyped belonged to a large pedigree, a family-based association test was also performed using the Family Based Association Test (FBAT) toolkit (v2.0.3) (http://www.hsph.harvard.edu/~fbat/fbat.htm) [34]. The FBAT toolkit improves upon the traditional transmission disequilibrium test (TDT) [70] by handling factors such as missing parents, additional family members, different genetic models and phenotypes, as well as controlling for false positive associations due to population structure [34]. A Bonferroni correction for multiple comparisons was implemented using JMP Genomics (SAS; Cary, NC) with adjusted P-values less than 0.05 considered significant.

### Evaluation of discrimination potential of the marker

Receiver operator characteristic curve analysis was performed using Stata v10 (www.stata.com).

### Targeted sequence capture

A 12 MB genomic region of interest was targeted using a custom array designed and manufactured by Roche NimbleGen (Madison, WI). 19,720 tiled probes approximately 60–90 bp in length covered approximately 96% of the targeted bases between 34,000,000–46,000,000 bp on CFA4. Unique probes were determined using the Sequence Search and Alignment by Hashing Algorithm (SSAHA) [71]. Targeted bases not captured were due to SSAHA's inability to determine valid probes possibly resulting from non-unique sequence, repetitive sequence, homopolymer runs or ambiguous bases.

Three micrograms of genomic DNA from three cases and three controls was used to prepare libraries for sequencing. DNA samples were fragmented by sonication (Covaris; Woburn, MA) and the fragments end-repaired, A-tailed, and ligated to indexing oligonucleotide adapters using NEBNext reagents (New England Biolabs; Ipswich, MA). Indexing adapters were provided by the Broad Institute (Cambridge, MA). The indexed DNA fragments were enriched for by PCR using AccuPrime (Life Technologies; Grand Island, NY) and Phusion (New England Biolabs, Ipswich, MA) DNA polymerases. DNA purification was done using QIAquick and MinElute PCR purification kits (Qiagen; Valencia, CA). Size selection and purification was done using Agencourt AMPure XP beads (Beckman Coulter; Beverly, MA). Analysis of DNA libraries was done using the Agilent Bioanalyzer DNA 1000 (Agilent Technologies; Santa Clara, CA) and Quant-iT dsDNA HS assay (Life Technologies, Grand Island, NY). Libraries were hybridized onto the array using a NimbleGen hybridization system (Roche NimbleGen; Madison, WI) at 42°C for 70 hours. Arrays were washed and samples eluted using NimbleGen's elution system (Roche NimbleGen; Madison, WI). Post-capture amplification was done using the primers 5′-AAT GAT ACG GCG ACC ACC GAG-3′and 5′-GAA GCA GAA GAC GGC ATA CGA-3′ with Phusion (New England Biolabs; Ipswich, MA) and AccuPrime (Life Technologies; Grand Island, NY) enzymes. Quantitative PCR (qPCR) using QuantiFast SYBR Green PCR kit (Qiagen; Valencia, CA) was done to estimate relative fold-enrichment of the target region (Table S5).

Paired-end sequencing was done on Illumina's Hi-Seq 2000 machine (Illumina; San Diego, CA) at the Broad Institute (Cambridge, MA).

### Sequencing data analysis

Sequence reads were aligned to the canine reference genome CanFam2 using the Burrows-Wheeler Aligner (BWA) [72]. SNPs and insertion/deletions (indels) were determined using the Genome Analysis Toolkit (GATK; http://www.broadinstitute.org/gatk/) [35]. Sequence data was processed according to the current best practices for data analysis found on the online GATK Wiki (http://www.broadinstitute.org/gsa/wiki/index.php/Main_Page) and included; initial SNP and indel calling, correcting alignment errors due to indels and inaccurate base quality scores, SNP and indel calling after the realignment and recalibration, and filtering the data using standard filtering parameters. Sequence and variants were viewed with the Integrative Genomic Viewer (IGV) [73]. Variants were identified in which all three affected dogs were homozygous for the non-reference allele, the two control carriers were heterozygous and the final control dog was either heterozygous or homozygous for the reference allele. Variants resulting in amino acid changes of coding regions of the genome were considered potential causative mutations for hereditary ataxia.

### Re-sequencing and genotyping variants

Variants considered as potential causative mutations were genotyped via Sanger sequencing in Old English Sheepdogs and additional dog breeds. The *RAB24* gene was also sequenced in affected dogs of breeds in which the A>C *RAB24* variant was not present. Primers were designed using Primer3 (http://frodo.wi.mit.edu/) [74] or NCBI's Primer-BLAST (http://www.ncbi.nlm.nih.gov/tools/primer-blast/) [75] (Table S6). PCR reactions included: 10× Buffer B (Thermo Fisher Scientific; Waltham, MA), 25 mM MgCl_2_ (Thermo Fisher Scientific; Waltham, MA), 10× MasterAmp (Epicentre Biotechnologies Madison, WI), 25 mM dNTPs (Apex BioResearch Products, San Diego, CA), 10 µM forward and reverse primer (IDT; Coralville, IA), Taq DNA polymerase (Apex BioResearch Products; San Diego, CA) and molecular grade water to a volume of 27 µL. DNA amounts varied from approximately 25 ng to 100 ng per reaction. Thermocycler conditions varied by primer set (Table S7). PCR products were purified using either Agencourt AMPure XP kit (Beckman Coulter; Beverly, MA), QIAquick PCR Purification Kit (Qiagen; Valencia, CA), or MinElute PCR Purification Kit (Qiagen; Valencia, CA). The sequencing of each purified PCR product (20–40 ng/µl for 8 µl) was carried out using the same forward and reverse primers used for PCR. Sequencing was performed by Eurofins MWG Operon (Huntsville, AL) following the standard BigDye Terminator v3.1 manufacture's protocol (Applied Biosystems; Foster, CA) with capillary electrophoresis carried out on the ABI 3730×l DNA Analyzer (Applied Biosystems; Foster, CA).

### RNA sequencing and qRT-PCR

Sections of cerebellum were collected from euthanized affected Old English Sheepdogs (n = 2), affected Gordon Setters (n = 2) and normal beagles (n = 2), and the tissue immediately frozen in liquid nitrogen. RNA was extracted from approximately 30 mg of the frozen tissue using Qiagen's RNeasy Mini Kit (Qiagen; Valencia, CA) and concentrations measured using a ND-1000 UV-Vis spectrophotometer (Thermo Scientific; Wilmington, DE).

Reverse transcription was performed using the Stratagene AffinityScript Multiple Temperature cDNA synthesis kit (Agilent Technologies; Santa Clara, CA) and 0.5 µg oligo(dT) and 0.2 µg random primers. Thermocycler conditions were set to 25°C for 10 minutes, 42°C for 5 minutes, 25°C for 60 minutes, and 72°C for 15 minutes.

Primers to amplify the mRNA were designed using Primer3 (http://frodo.wi.mit.edu/) [74], with forward primer 5′-CGTGTCTCCAGGCGTAGC-3′ and reverse primer 5′-ACTGGGGGTAGCTCAGAC-3′. The approximately 850 bp PCR product was excised from an ethidium bromide stained 2% agarose gel and cleaned using the QIAquick Gel Extraction Kit (Qiagen; Valencia, CA). The cDNA of an affected Old English Sheepdog and Gordon Setter as well as a normal beagle were sequenced using the same forward and reverse PCR primers. Sequencing was performed as was done for genotyping the variants.

Quantitative PCR was performed using Applied Biosystems' StepOne Plus instrument and Invitrogen's Power SYBR Green Master Mix (Life Technologies; Grand Island, NY). *RAB24* primers were designed using Primer3 (http://frodo.wi.mit.edu/) [74] and PerlPrimer (http://perlprimer.sourceforge.net/) [76]. The housekeeping gene *RPS19* (primers published [77]) was used for normalization. 20 µl reaction volumes containing 200 nM primers were done in triplicate. Cycling conditions consisted of 95°C for 10 minutes followed by 40 cycles of 95°C for 15 seconds and 60°C for 1 minute with a melt curve performed at completion. Reaction efficiencies were calculated using a 6 point 2∶1 dilution series of 100 ng cDNA. *RAB24* and *RPS19* efficiencies were estimated to be 90% and 88% respectively with both r^2^ values of 0.999. Relative expression differences were calculated using the ΔΔCt-method and statistical significance determined using Microsoft Excel (2010) to perform an unpaired t-test on the relative differences between cases and controls.

### Histopathology and immunohistochemistry

For histopathology, tissues were fixed in 10% neutral buffered formalin, embedded in paraffin, sectioned to five micrometers and stained with hematoxylin and eosin, PAS, Luxol Fast Blue/Cresyl Violet and Bielschowsky Silver Stain. Immunohistochemistry for GFAP, ubiquitin and Rab24 was performed on formalin fixed paraffin embedded brain tissue. Rabbit polyclonal antibodies were used for all three antigens purchased from Dako (GFAP), Santa Cruz Biotechnology (ubiquitin) and Proteintech Group Inc. (Rab24). 5 µm sections were cut, deparaffinized and rehydrated. GFAP and ubiquitin immunostaining were performed using the Dako Autostainer. Rab24 immunostaining was performed manually. The autostaining method included a five-minute retrieval for GFAP using Dako Proteinase K (Dako; Carpinteria, CA), followed by blocking of endogenous peroxidase activity using 3% hydrogen peroxide for 10 minutes. The primary antibody was applied (concentrations and incubation time and conditions are given in Table S8), and the product was visualized by incubation with Dako Envision and rabbit polymer (Dako; Carpinteria, CA) for 30 minutes followed by application of DAB for five minutes. Slides were counterstained with hematoxylin, dehydrated through sequential alcohol immersion to xylene and cover-slipped. Rab24 immunostaining involved a citrate retrieval using a Pascal Pressurized Heating Chamber at 120°C, followed by blocking of endogenous peroxidase activity using 3% hydrogen peroxide for 10 minutes. A protein block was then performed using normal goat serum (protein block/normal goat serum, Biogenex; Fremont, CA) applied for 20 minutes, followed by incubation with rabbit anti-Rab24 at a concentration of 1∶50 for 1 hour at room temperature. Slides were washed with phosphate buffered saline (PBS) then biotinylated goat anti-rabbit antibody (Link; Biogenex, Fremont, CA) was applied diluted to 1∶20 and incubated for 20 minutes at room temperature. Following washing, peroxidase conjugated streptavidin (Label; Biogenex, Fremont, CA) was applied and incubated for a further 20 minutes at room temperature. The product was developed under the microscope using DAB for about one minute to an appropriate level of staining. The slides were counterstained with hematoxylin, dehydrated through sequential alcohol immersion to xylene and cover-slipped.

### Transmission electron microscopy

Sections were prepared for transmission electron microscopy from freshly harvested cerebellar tissue as described previously [11]. Samples of cerebellar cortex were cut into approximately 1 mm^2^ cubes and fixed in McDowell's and Trump's 4F:1G fixative prior to incubation in 1% osmium tetroxide for one hour. Following this period of fixation, samples were placed in a 1∶1 mixture of Spurr resin (EMS Spurr resin kit 14300, Hatfield, PA) and acetone for 30 minutes and then placed in 100% resin changed three times at two hourly intervals. The final change of resin was polymerized at 70°C for eight hours. Semi-thin sections (0.25 µm) were cut, stained with 1% toluidine blue-O in 1% sodium borate and used to identify areas of interest. Ultrathin sections (70–90 nm) of these areas were stained with methanolic uranyl acetate (EMS 22400, Hatfield, PA), followed by lead citrate, and examined by TEM (FEI/Philips EM208S TEM, Oregon, USA). Reagents were obtained from Fisher Scientific (Pittsburgh, PA) unless otherwise indicated.

### Ethics statement

All animals evaluated in this study were privately owned pets. DNA samples were obtained with client consent and approval of the Institutional Animal Use and Care Committee or from DNA banks. Euthanasia and necropsies were performed at the owners' request.

## Supporting Information

Figure S1 The SNP genotypes of Gordon Setters and Old English Sheepdogs on CFA4 demonstrating a shared region of homozygosity in affected dogs from both breeds extending from 39,245,536 bp to 41,172,873 bp. Each row is a different individual and each column is a different SNP. Solid green (C), blue (G) and red (A) boxes indicate homozygosity for the respective nucleotides while yellow boxes indicate heterozygosity.
(TIFF)

Table S1 Details of six exonic non-synonymous SNPs detected by next generation sequencing.
(DOCX)

Table S2 Old English Sheepdog genotypes of six exonic SNPs. The case that exhibits the control genotype (A/A and C/C for *RAB24 and NSD1*) was removed from the dataset as its SNP genotypes were not consistent with those of its parents.
(DOCX)

Table S3 Additional dog breeds genotyped on *RAB24* and *NSD1*. None of the dogs exhibited signs of cerebellar disease.
(DOCX)

Table S4 Human genes located in the region of shared homozygosity between affected Old English Sheepdogs and Gordon Setters.
(DOCX)

Table S5 Primer sets used for qPCR during targeted sequence capture.
(DOCX)

Table S6 Primer sets to investigate the six exonic SNP variants further and for Sanger sequencing of *RAB24*.
(DOCX)

Table S7 Thermocycler conditions for genotyping variants and Sanger sequencing of *RAB24*.
(DOCX)

Table S8 Antibody concentrations and conditions for immunohistochemistry of formalin fixed, paraffin embedded tissue.
(DOCX)

Video S1 Videotape of the gait of a three-year-old female, affected Old English Sheepdog. She exhibits a hypermetric gait, particularly in her thoracic limbs, and a pronounced truncal sway.
(MOV)
