# Supplementary figures and images for: Canine Hereditary Ataxia in Old English Sheepdogs and Gordon Setters Is Associated with a Defect in the Autophagy Gene Encoding RAB24
Source: PLoS Genet. 2014 Feb 6;10(2):e1003991. doi: 10.1371/journal.pgen.1003991 (PMC3916225; doi:10.1371/journal.pgen.1003991)

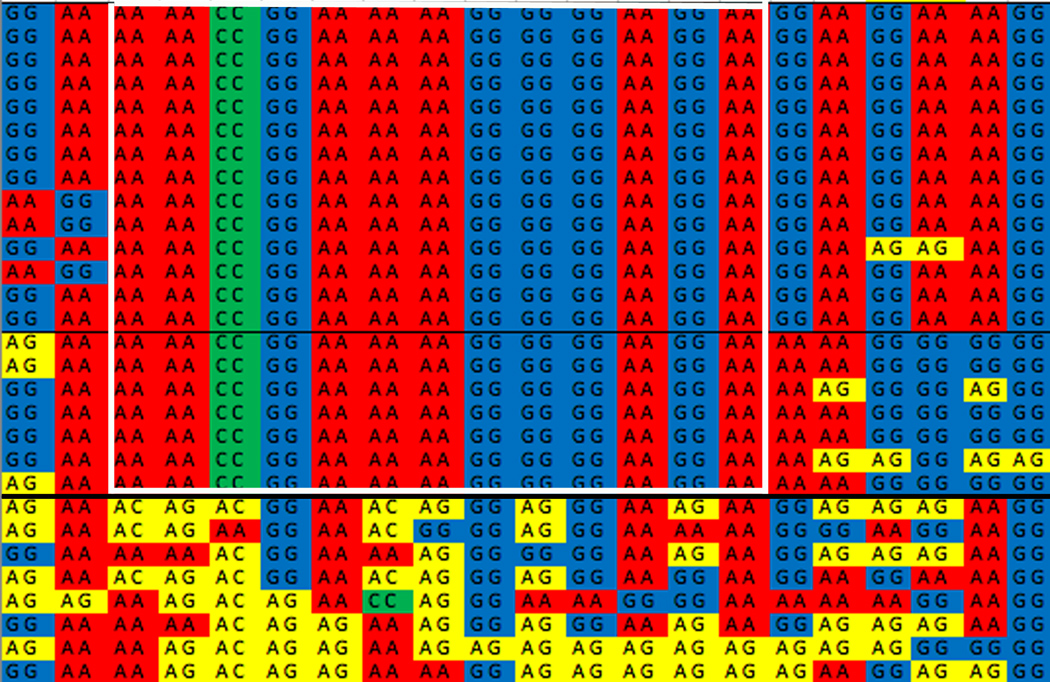

Supplement: Figure S1 — The SNP genotypes of Gordon Setters and Old English Sheepdogs on CFA4 demonstrating a shared region of homozygosity in affected dogs from both breeds extending from 39,245,536 bp to 41,172,873 bp. Each row is a different individual and each column is a different SNP. Solid green (C), blue (G) and red (A) boxes indicate homozygosity for the respective nucleotides while yellow boxes indicate heterozygosity. (TIFF) [file pgen.1003991.s001.tiff]
