# Supplementary material for: Canine Hereditary Ataxia in Old English Sheepdogs and Gordon Setters Is Associated with a Defect in the Autophagy Gene Encoding RAB24
Source: PLoS Genet. 2014 Feb 6;10(2):e1003991. doi: 10.1371/journal.pgen.1003991 (PMC3916225; doi:10.1371/journal.pgen.1003991)
Supplement: Table S1 — Details of six exonic non-synonymous SNPs detected by next generation sequencing. (DOCX) [file pgen.1003991.s002.docx]

**Table S1**

| **SNP position (bp,** **CanFam 2)** | **Gene Abr.** | **Gene** | **Reference allele** | **Alternate allele** | **Amino acid change** |
| --- | --- | --- | --- | --- | --- |
| 35,707,658 | *RGR* | Retinal G protein coupled receptor | G | T | Trp>Leu |
| 39,246,812 | *RAB24* | Member Ras oncogene family | A | C | Gln>Pro |
| 39,319,721 | *NSD1* | Nuclear receptor binding SET domain protein 1 | C | G | Lys>Asn |
| 39,929,599 | *GPRIN1* | G protein regulated inducer of neurite outgrowth 1 | G | A | Arg>Lys |
| 39,929,601 | *GPRIN1* | G protein regulated inducer of neurite outgrowth 1 | G | A | Ala>Thr |
| 39,940,607 | *CDHR2* | Cadherin-related family member 2 | C | G | Arg>Ser |

**Table S1:** Details of six exonic non-synonymous SNPs detected by next generation sequencing
