# Supplementary material for: Canine Hereditary Ataxia in Old English Sheepdogs and Gordon Setters Is Associated with a Defect in the Autophagy Gene Encoding RAB24
Source: PLoS Genet. 2014 Feb 6;10(2):e1003991. doi: 10.1371/journal.pgen.1003991 (PMC3916225; doi:10.1371/journal.pgen.1003991)
Supplement: Table S2 — Old English Sheepdog genotypes of six exonic SNPs. The case that exhibits the control genotype (A/A and C/C for RAB24 and NSD1) was removed from the dataset as its SNP genotypes were not consistent with those of its parents. (DOCX) [file pgen.1003991.s003.docx]

**Table S2**

| **SNP position (bp, CanFam 2)** | **Gene** | **Case phenotype** | **Case genotype** | **Control phenotype** | **Control genotype** |
| --- | --- | --- | --- | --- | --- |
| 35,707,658 | *RGR* | 5 | 5-T/T | 15 | 2-T/T, 8-G/T, 5-G/G |
| 39,246,812 | *RAB24* | 16 | 15-C/C, 1-A/A | 100 | 57-A/C, 43-A/A |
| 39,319,721 | *NSD1* | 16 | 15-G/G, 1-C/C | 97 | 53-C/G, 44-C/C |
| 39,929,599 | *GPRIN1* | 9 | 9-A/A | 42 | 15-A/A, 19-G/A, 8-G/G |
| 39,929,601 | *GPRIN1* | 9 | 9-AA | 42 | 16-A/A, 16-G/A,10-G/G |
| 39,940,607 | *CDHR2* | 7 | 7-GG | 17 | 4-G/G, 12-C/G, 4-C/C |

**Table S2**: Old English Sheepdog genotypes of six exonic SNPs. The case that exhibits the control genotype (A/A and C/C for *RAB24 and NSD1)* was removed from the dataset as its SNP genotypes were not consistent with those of its parents.
