# Supplementary material for: Canine Hereditary Ataxia in Old English Sheepdogs and Gordon Setters Is Associated with a Defect in the Autophagy Gene Encoding RAB24
Source: PLoS Genet. 2014 Feb 6;10(2):e1003991. doi: 10.1371/journal.pgen.1003991 (PMC3916225; doi:10.1371/journal.pgen.1003991)
Supplement: Table S3 — Additional dog breeds genotyped on RAB24 and NSD1. None of the dogs exhibited signs of cerebellar disease. (DOCX) [file pgen.1003991.s004.docx]

**Table S3**

| **Breed** | **No. dogs genotyped on *RAB24* SNP** | **No. dogs genotyped on *NSD1* SNP** |
| --- | --- | --- |
| Airedale Terrier | 1 |  |
| Akita | 1 |  |
| American Staffordshire Terrier | 10 | 7 |
| Australian Shepherd | 7 |  |
| Beagle | 4 |  |
| Berger Picard | 1 |  |
| Border Collie | 5 |  |
| Boston Terrier | 3 |  |
| Boxer | 8 | 7 |
| Brittany Spaniel | 1 |  |
| Cairn Terrier | 4 |  |
| Chihuahua | 5 |  |
| Chow Chow | 1 |  |
| Cocker Spaniel | 7 |  |
| Dachshund | 8 |  |
| Dalmatian | 1 |  |
| Doberman Pinscher | 8 | 6 |
| Dogue De Bordeaux | 1 |  |
| English Cocker Spaniel | 1 |  |
| English Pointer | 1 |  |
| English Setter | 3 |  |
| English Springer Spaniel | 1 |  |
| Golden Retriever | 5 |  |
| Greyhound | 6 |  |
| German Shepherd | 11 | 10 |
| Irish Setter | 9 |  |
| King Charles Spaniel | 14 |  |
| Labrador Retriever | 12 | 7 |
| Mastiff | 1 |  |
| Pekingese | 3 |  |
| Pembroke Welsh Corgi | 2 |  |
| Pug | 1 |  |
| Rhodesian Ridgeback | 2 |  |
| Scottish Deerhound | 7 |  |
| Scottish Terrier | 10 | 10 |
| Shetland Sheepdog | 1 |  |
| Shiba-Inu | 1 |  |
| Shih Tzu | 3 |  |
| Siberian Husky | 4 |  |
| Spitz | 2 |  |
| Standard Poodle | 8 | 7 |
| Weimaraner | 2 |  |
| West Highland White Terrier | 11 | 10 |
| Wheaton Terrier | 1 |  |

**Table S3:** Additional dog breeds genotyped on *RAB24* and *NSD1*. None of the dogs exhibited signs of cerebellar disease.
