# Supplementary material for: Canine Hereditary Ataxia in Old English Sheepdogs and Gordon Setters Is Associated with a Defect in the Autophagy Gene Encoding RAB24
Source: PLoS Genet. 2014 Feb 6;10(2):e1003991. doi: 10.1371/journal.pgen.1003991 (PMC3916225; doi:10.1371/journal.pgen.1003991)
Supplement: Table S4 — Human genes located in the region of shared homozygosity between affected Old English Sheepdogs and Gordon Setters. (DOCX) [file pgen.1003991.s005.docx]

**Table S4**

| **Gene** | **Location (bp, CanFam 2)** |
| --- | --- |
| *PRELID1* | 39242961-39246256 |
| *RAB24* | 39246277-39248659 |
| *NSD1* | 39249489-39403941 |
| *FGFR4* | 39434643-39442824 |
| *ZNF346* | 39461568-39494567 |
| *UIMC1* | 39545843-39657854 |
| *HK3* | 39667276-39680511 |
| *UNC5A* | 39680507-39742553 |
| *TSPAN17* | 39876118-39885089 |
| *EIF4E1B* | 39885950-39890054 |
| *SNCB* | 39901120-39910339 |
| *GPRIN1* | 39927715-39931812 |
| *CDHR2* | 39931923-39958098 |
| *RNF44* | 39982637-39993392 |
| *FAF2* | 40010626-40083751 |
| *TRIP10* | 40104034-40104850 |
| *CLTB* | 40108059-40128212 |
| *HIGD2A* | 40130554-40131209 |
| *NOP16* | 40131462-40135664 |
| *ARL10* | 40139780-40147536 |
| *KIAA1191* | 40159263-40173432 |
| *SIMC* | 40173581-40250788 |
| *THOC3* | 40323805-40344171 |
| *MANBAL* | 40436862-40437294 |
| *CPLX2* | 40395873-40480130 |
| *SELK* | 40565418-40566002 |
| *HRH2* | 40575739-40599575 |
| *Sfxn1* | 40678995-40699631 |
| *DRD1* | 40743436-40746624 |

**Table S4:** Human genes located in the region of shared homozygosity between affected Old English Sheepdogs and Gordon Setters.
