# Supplementary material for: Canine Hereditary Ataxia in Old English Sheepdogs and Gordon Setters Is Associated with a Defect in the Autophagy Gene Encoding RAB24
Source: PLoS Genet. 2014 Feb 6;10(2):e1003991. doi: 10.1371/journal.pgen.1003991 (PMC3916225; doi:10.1371/journal.pgen.1003991)
Supplement: Table S5 — Primer sets used for qPCR during targeted sequence capture. (DOCX) [file pgen.1003991.s006.docx]

**Table S5**

|  | **Forward Primer** | **Reverse Primer** |
| --- | --- | --- |
| On target 1 | 5’-TCATCTCAGCGTTCCACAAG-3’ | 5’-TGTCATTAGAGGGAGGCAAG-3’ |
| On target 2 | 5’-CAAGGGGAAGAATGGAAAGA-3’ | 5’-TAAAGGAGAGTGGGCAGGTT-3’ |
| On target 3 | 5’-AAGGTGAAGGCAAGCAATGT-3’ | 5’-TGAGGAGCAGGTGTTGTTCT-3’ |
| Off target 1 | 5’-TGGCATGTGTCTCCTTTCAA-3’ | 5’-TCCTGTCCCGTGATTTACAG-3’ |
| Off target 2 | 5’-ATGGGTCTGGGATGAAGGAT-3’ | 5’-AGTCTGAGGGCCATTGAACA-3’ |
| Off target 3 | 5’-TGCAATTTCCCACATGCTC-3’ | 5’-TTGAGACAGATGGTATGCAGTG-3’ |

**Table S5**: Primer sets used for qPCR during targeted sequence capture.
