# Supplementary material for: Canine Hereditary Ataxia in Old English Sheepdogs and Gordon Setters Is Associated with a Defect in the Autophagy Gene Encoding RAB24
Source: PLoS Genet. 2014 Feb 6;10(2):e1003991. doi: 10.1371/journal.pgen.1003991 (PMC3916225; doi:10.1371/journal.pgen.1003991)
Supplement: Table S6 — Primer sets to investigate the six exonic SNP variants further and for Sanger sequencing of RAB24. (DOCX) [file pgen.1003991.s007.docx]

**Table S6**

| **Target** | **Forward** | **Reverse** |
| --- | --- | --- |
| *RGR* | 5’-CCCTTGTGTCACGACAGGT-3’ | 5’-CTCCTCTGCGGTGACAGG-3’ |
| *RAB24* | 5’-GTGAGGCAGCCCAGACAG-3’ | 5’-CCAGGGGACTTACCCAAATA-3’ |
| *NSD1* | 5’-GAGCTGCCTGAAACCAAGAC-3’ | 5’-TCCCGTTGTAGTGGAACCTC-3’ |
| *GPRIN1* | 5’-TGGCATGTGTCTCCTTTCAA-3’ | 5’-TCCTGTCCCGTGATTTACAG-3’ |
| *CDHR2* | 5’-AGCTGAGAAATGGTATCCAC-3’ | 5’-GGGCTTGGTGCTAGAGTCTG-3’ |
| Upstream/ Exon 1 | 5’-CCCCTCACCCTTTACACTCG-3’ | 5’-CGCCAGTCACGTTCTGATAG-3’ |
| Exon 2-3 | 5’-GGCCCTATCAGAACGTGAGT-3’ | 5’-AGACACGCAGAGAACAACCA-3’ |
| Exon 4-5 | 5’-AAACAAGAGGGGGTGGATGCT-3’ | 5’-ATGGCAGCTAAGGCAGTTCAT -3’ |
| Exon 6-7 | 5’-CCCTTGGGAAGAGCTTCTGG-3’ | 5’-CTGTGGGGCTACAGGAAGTC-3’ |
| Exon8/Downstream | 5’-GGCCCCCTTCACGAGTTAC-3’ | 5’-GTGGGAAGTCAGGCCAGCCA-3’ |

**Table S6:** Primer sets to investigate the six exonic SNP variants further and for Sanger sequencing of *RAB24*.
