# Supplementary material for: Canine Hereditary Ataxia in Old English Sheepdogs and Gordon Setters Is Associated with a Defect in the Autophagy Gene Encoding RAB24
Source: PLoS Genet. 2014 Feb 6;10(2):e1003991. doi: 10.1371/journal.pgen.1003991 (PMC3916225; doi:10.1371/journal.pgen.1003991)
Supplement: Table S7 — Thermocycler conditions for genotyping variants and Sanger sequencing of RAB24. (DOCX) [file pgen.1003991.s008.docx]

**Table S7**

| **Sequencing of *RAB24*, *NSD1*, *CDHR2*, *GPRIN1*, and *RGR* SNPs** | | | |
| --- | --- | --- | --- |
| Step | Temperature ( °C) | Time (minutes) |  |
| 1 | 98 | 0:30 |  |
| 2 | 98 | 0:30 |  |
| 3 | 68 | 0:30 | Decrease by 1°C every cycle |
| 4 | 72 | 0:30 |  |
| 5 | Cycle to Step 2 for 13 more times | |  |
| 6 | 98 | 1:00 |  |
| 7 | 59 | 1:00 |  |
| 8 | 72 | 1:00 |  |
| 9 | Cycle to Step 6 for 40 more times | |  |
| 10 | 72 | 5:00 |  |
| 11 | 12 | Forever |  |
| **Sequencing of *RAB24* gene** | | | |
| 1 | 94 | 3:00 |  |
| 2 | 94 | 0:30 |  |
| 3 | 68 | 0:30 | Decrease by 1°C every cycle |
| 4 | 72 | 1:00 |  |
| 5 | Cycle to Step 2 for 5 more times | |  |
| 6 | 94 | 0:30 |  |
| 7 | 61.8 | 0:30 |  |
| 8 | 72 | 1:00 |  |
| 9 | Cycle to Step 6 for 30 more times | |  |
| 10 | 72 | 10:00 |  |
| 11 | 12 | Forever |  |

**Table S7:** Thermocycler conditions for genotyping variants and Sanger sequencing of *RAB24*.
