# Supplementary material for: Canine Hereditary Ataxia in Old English Sheepdogs and Gordon Setters Is Associated with a Defect in the Autophagy Gene Encoding RAB24
Source: PLoS Genet. 2014 Feb 6;10(2):e1003991. doi: 10.1371/journal.pgen.1003991 (PMC3916225; doi:10.1371/journal.pgen.1003991)
Supplement: Table S8 — Antibody concentrations and conditions for immunohistochemistry of formalin fixed, paraffin embedded tissue. (DOCX) [file pgen.1003991.s009.docx]

**Table S8**

| **Antibody** | **Concentration** | **Incubation time and conditions** |
| --- | --- | --- |
| Rabbit anti GFAP | 1:500 | 15 minutes RT |
| Rabbit anti ubiquitin | 1:50 | 30 minutes RT |
| Rabbit anti Rab24 | 1:50 | Overnight at 4 ^o^C |

**Table S8:** Antibody concentrations and conditions for immunohistochemistry of formalin fixed, paraffin embedded tissue.
